# Multimodal Imaging Nanoparticles Derived from Hyaluronic Acid for Integrated Preoperative and Intraoperative Cancer Imaging

**DOI:** 10.1155/2017/9616791

**Published:** 2017-09-11

**Authors:** William M. Payne, Tanner K. Hill, Denis Svechkarev, Megan B. Holmes, Balasrinivasa R. Sajja, Aaron M. Mohs

**Affiliations:** ^1^Department of Pharmaceutical Sciences, University of Nebraska Medical Center, Omaha, NE, USA; ^2^Department of Radiology, University of Nebraska Medical Center, Omaha, NE, USA; ^3^Department of Biochemistry and Molecular Biology, University of Nebraska Medical Center, Omaha, NE, USA; ^4^Fred and Pamela Buffett Cancer Center, University of Nebraska Medical Center, Omaha, NE, USA

## Abstract

Surgical resection remains the most promising treatment strategy for many types of cancer. Residual malignant tissue after surgery, a consequence in part due to positive margins, contributes to high mortality and disease recurrence. In this study, multimodal contrast agents for integrated preoperative magnetic resonance imaging (MRI) and intraoperative fluorescence image-guided surgery (FIGS) are developed. Self-assembled multimodal imaging nanoparticles (SAMINs) were developed as a mixed micelle formulation using amphiphilic HA polymers functionalized with either GdDTPA for *T*_1_ contrast-enhanced MRI or Cy7.5, a near infrared fluorophore. To evaluate the relationship between MR and fluorescence signal from SAMINs, we employed simulated surgical phantoms that are routinely used to evaluate the depth at which near infrared (NIR) imaging agents can be detected by FIGS. Finally, imaging agent efficacy was evaluated in a human breast tumor xenograft model in nude mice, which demonstrated contrast in both fluorescence and magnetic resonance imaging.

## 1. Introduction

Surgical resection remains the most promising treatment strategy for many forms of cancer. Augmenting the efficacy of surgical intervention in cancer treatment through technologies aiming to improve contrast, such as image-guided surgery, can improve the prognosis and outcome of cancer treatment [1–3]. Residual cancerous tissue after surgery contributes to tumor recurrence and is a limitation of surgical efficacy, reducing the chances for long-term survival. Because surgeons are limited by what they can see and feel [4], tumor recurrence resulting from positive margins and metastases presents an opportunity to increase survival [2]. Thus, complete removal of cancerous tissue during the initial surgical intervention is critical to improved prognosis.

Guidance during surgery for the detection of surgical margins and regional metastases is not easily accomplished using conventional imaging techniques, such as magnetic resonance imaging (MRI), positron emission tomography (PET), or single-photon emission computed tomography (SPECT) due to the necessity of large or not easily modularized instrumentation [4]. Fluorescence image-guided surgery (FIGS) has recently emerged as a promising technique for intraoperative guidance. Contrast between malignant and healthy tissue requires higher signal in the tumor than the surrounding, healthy, or benign tissue. As reported by us [1, 5, 6] and others [3, 7], the use of near infrared fluorescence contrast agents, both FDA-approved (i.e., indocyanine green (ICG)) and experimental, can guide surgeons in real-time, delineating tumor boundaries and improving outcome [2, 3].

Integrating a preoperative imaging technique such as MRI or CT with FIGS could potentially improve surgical resection efficacy even more by defining margins for preoperative planning or further guiding surgeons to regional metastases [8]. Contrast agents must have suitable pharmacokinetic parameters, limited toxicity, and high sensitivity. To further improve the efficacy of FIGS, nanoparticle formulations are often used to specifically target or achieve higher uptake in cancerous tissue [9–11]. Nanoparticulate imaging agents have demonstrated superiority over combinations of small molecules [12] and are also more versatile, allowing for the incorporation of targeting moieties, drugs, and a variety of chemical modification into the imaging agent. While inorganic nanoparticles have been used for various imaging combinations, polymeric nanoparticles benefit from improved biocompatibility [12, 13].

Modification of naturally occurring or biocompatible polymers with moieties for contrast, such as radioisotopes, fluorophores, or gadolinium can produce stable, biocompatible, multimodal imaging agents. We have recently reported on the use of hyaluronic acid- (HA-) derived nanoparticles for imaging [5, 6, 14] and drug delivery [15]. To date, however, these HA nanostructures were primarily developed for intraoperative imaging. In this study we report the optimization of an HA-based nanoparticle formulation for integrated preoperative MRI and intraoperative near infrared fluorescence imaging, termed self-assembled multimodality imaging nanoparticles (SAMINs). HA is a naturally occurring glycosaminoglycan that, when modified with hydrophobic moieties, can self-assemble into nanoparticles [16–18]. By conjugation of a fluorescent dye and Gd(III), the modified HA polymers provide contrast for both fluorescence and MR imaging, respectively. Furthermore, the ratio of fluorophore to paramagnetic agent can be tuned for optimal fluorescence and MR contrast.

Herein, we synthesize a new formulation of HA-based contrast agents. We then demonstrate efficacy in vitro by characterizing the contrast obtained in different tissue phantom models and characterizing nonmalignant cell response to the contrast agents. Finally, the SAMINs are shown to produce contrast in MR and fluorescence imaging, providing proof-of-concept for integrated preoperative and intraoperative imaging. We demonstrate that HA-based nanoparticles incorporating both gadolinium and a fluorophore can provide contrast enhancement for both imaging modalities, with the goal of providing better surgical guidance for tumor resection and ultimately improving prognosis.

## 2. Materials and Methods

1-Pyrenebutyric acid, diethylenetriaminepentaacetic acid (DTPA), 1,3-diaminopropane, N-hydroxysuccinimide, 1-ethyl-3-(3-dimethylaminopropyl)carbodiimide (EDC), gadolinium (III) chloride, and xylenol orange were purchased from Sigma-Aldrich (St. Louis, MO). Dimethyl sulfoxide (DMSO), methanol, N,N-dimethylformamide (DMF), concentrated hydrochloric acid (HCl), concentrated nitric acid, 1x phosphate-buffered saline (PBS), and molecular sieves (type 3A) were purchased from Fisher Scientific (Pittsburgh, PA). Ethanol was purchased from UNMC internal supply. Sodium hyaluronate (HA) was purchased from Lifecore Biomedical (Chaska, MN). Matrigel was purchased from BD Biosciences (San Jose, CA). Cy7.5-amine was purchased from Lumiprobe Corporation (Hallandale Beach, FL).

### 2.1. Conjugation of Aminopropyl-1-pyrenebutanamide to HA

Aminopropyl-1-pyrenebutanamide (PBA) was synthesized from 1-pyrenebutyric acid as previously described [6, 14]. PBA-modified HA (HA-PBA) was synthesized by dissolving 90–95 mg HA (*M*_*N*_ = 10–20 kDa) in 1 : 1 DMF : H_2_O. NHS and EDC were added to the HA solution in 10-fold molar excess and allowed to mix for 30 minutes. PBA (5–10 wt%) was first dissolved in 5 mL DMF, then added dropwise to the HA solution, and allowed to react for 24 h at room temperature. The reaction mixture was then dialyzed against 1 : 1 EtOH : H_2_O for 24 h (4 exchanges) and water alone for 48 h (8 exchanges). The HA-PBA product was then lyophilized and stored at −20°C.

### 2.2. Conjugation of Cy7.5-Amine to PBA-Modified HA

Cy7.5-amine was conjugated to the amphiphilic PBA-modified HA as previously described by Kelkar et al. [6]. Briefly, PBA-HA (18.0 mg) was dissolved in 10 mL of 1 : 1 DMSO : H_2_O. NHS and EDC were added to the PBA-HA solution and stirred for 30 minutes at room temperature to activate the carboxylic acid groups of HA. A stock Cy7.5-amine solution in DMSO was prepared and added dropwise to the HA reaction solution for a total of 2.0 mg Cy7.5. The reaction was covered to protect from light and allowed to proceed for 24 h under constant stirring at room temperature. The product was purified through dialysis against ultrapure water for 24–36 h (8 exchanges). After dialysis, any remaining excess dye was removed with PD10 desalting columns using ultrapure water as the mobile phase. Finally, the HA-PBA-Cy7.5 product was lyophilized and stored at −20°C, and will be referred to as HA-Cy.

### 2.3. Synthesis of Paramagnetic Amphiphilic HA

Addition of gadolinium to amphiphilic HA-PBA was accomplished by first conjugating DTPA to HA via dianhydride hydrolysis and coordination with Gd^3+^ was achieved based on a method reported by Moon et al. [19]. Briefly, DMSO was first dried over molecular sieves to remove any water. HA-PBA (50 mg) was then dissolved in the dry DMSO over 24 h, with bath sonication to assist dissolution if needed. The solution remained cloudy but would become transparent after reaction with DTPA dianhydride. After the HA-PBA was sufficiently dissolved, DTPA dianhydride (25 mg, 0.07 mmol) was dissolved in 10 mL of dry DMSO and added dropwise to the HA-PBA solution. The reaction was then allowed to proceed at room temperature for 48 h. The HA-PBA-DTPA product was then purified by dialysis against ultrapure water for 24–36 h (8 exchanges) and lyophilized. To synthesize paramagnetic HA derivatives, Gd^3+^ was complexed with the DTPA moieties on HA-PBA-DTPA. First, HA-PBA-DTPA (20 mg) was dissolved in 20 mL of 1 : 1 DMSO : H_2_O and titrated to pH 7 with 10% NaOH solution. Next, GdCl_3_ (10.0 mg, 0.038 mmol) was dissolved in 5 mL of ultrapure water and added dropwise to the HA solution. The reaction was then allowed to proceed for 24 h under constant stirring, and pH was periodically checked and titrated to pH 7 with 10% NaOH or 10% HCl. Paramagnetic HA was purified by dialysis against ultrapure water over 24–36 h (8 exchanges), and any remaining free Gd^3+^ was removed with PD10 desalting columns using ultrapure water as the mobile phase. Finally, the paramagnetic HA was lyophilized and stored at −20°C.

### 2.4. Characterization of Paramagnetic HA

The addition of DTPA to amphiphilic HA-PBA was confirmed through infrared spectroscopy, using the procedure described by Moon et al. [19] Modified and unmodified (control) samples of HA powder were analyzed on a Perkin Elmer IR spectrometer. Gadolinium content of paramagnetic HA was determined by spectrophotometric colorimetry and inductively coupled plasma mass spectrometry (ICP-MS). The colorimetric assay was performed with a standard curve using xylenol orange as an indicator of free gadolinium after reaction completion, using a previously published protocol [20]. Briefly, a standard curve of 15 *µ*M xylenol orange in acetate buffer with a concentration of gadolinium ranging from 10 to 100 *µ*M was constructed. Unknown (experimental) gadolinium concentration remaining after the reaction could then be determined through spectrophotometry and back calculated to obtain the molar content of gadolinium per gram of HA conjugate. ICP-MS was used to confirm colorimetric measurements by first digesting the purified paramagnetic HA product with nitric acid and then providing the sample to the UNMC nanomaterial characterization core for ICP-MS analysis. The gadolinium content was then used to find the molar content of gadolinium per gram of paramagnetic HA conjugate. After determining the gadolinium content in the paramagnetic HA sample, *T*_1_-relaxivity experiments were performed on a 7 T/16 cm Bruker PharmaScan (Bruker; Ettlingen, Germany) preclinical MRI scanner operating on Paravision 5.1 software. Relaxivity image data were acquired using RAREVTR (Rapid Acquisition with Relaxation Enhancement (RARE) with variable repetition time) sequence with ten repetition (*T*_*R*_) times (10000, 5000, 3000, 1500, 1200, 800, 500, 450, 400, and 300 ms) and an echo time (*T*_*E*_) of 12.76 ms. Eleven slices (1 mm slice thickness) with image matrix size of 128 × 128 and field of view (FOV) of 30 mm × 30 mm were acquired for a total acquisition time of 9 min and 17 sec. *T*_1_ maps were generated using in-house developed computer program written in Interactive Data Language (IDL; Exelis Visual Information Solutions; McLean, VA, USA). A range of concentrations of paramagnetic HA, measured by gadolinium concentration, were analyzed to determine relaxivity.

### 2.5. Characterization of HA-Derived Nanoparticles

Nanoparticles were formed by self-assembly after dissolving the freeze-dried HA conjugates, that is, paramagnetic HA alone, fluorescent HA alone, or varying ratios of the paramagnetic and fluorescent HA conjugates, in ultrapure water. Except for the experiments investigating the formation of multimodal nanoparticles over time, nanoparticle samples were equilibrated for 4 h and filtered through a 0.45 *µ*m syringe filter prior to performing any measurements. Particle size, polydispersity index (PDI), and zeta potential were determined using a Malvern ZetaSizer ZS90 (Malvern Instruments; Malvern, UK). Transmission electron microscope (TEM) images were obtained using a FEI Tecnai G2 Spirit TEM (FEI; Hillsboro, Oregon) available in UNMC's electron microscopy core facility. Prior to TEM imaging, nanoparticles (concentration 1.0 mg/mL in ultrapure water) were placed on a formvar/silicone monoxide coated 200 mesh copper grids and allowed to adhere for approximately 2 minutes, NanoVan negative stain was applied for 30 seconds, and the sample was blotted to remove excess solvent or material.

### 2.6. Formulation Optimization

The ratio of HA-Cy to HA-Gd was optimized by comparing the fluorescence intensity to gadolinium content. Spectroscopic and imaging experiments were performed with a range of formulations to determine the optimal optical properties for imaging. Briefly, a series of nanoparticles with varying concentration of HA-Cy7.5 were prepared and characterized using a UV-Vis spectrophotometer (Thermo Fisher Scientific; Waltham, MA), a Fluoromax fluorometer (Horiba; Kyoto, Japan), a Pearl Trilogy Small Animal Imaging System (LI-COR; Lincoln, NE), and a custom-made fluorescence image-guided surgery system (FIGSS), previously described elsewhere [21, 22]. The appropriate mass of paramagnetic HA needed to yield a concentration of 100 *µ*M Gd^3+^ (the clinically ideal concentration for imaging [23]) was prepared as a stock solution, and varying concentration of HA-Cy7.5 was added to obtain a concentration ratio range from 0.8 to 80 Gd^3+^ ions to Cy7.5 molecules (referred to as Gd : Cy7.5 ratio hereon), and optical and magnetic properties were measured.

### 2.7. Cytotoxicity of SAMINs

Cytotoxicity was assessed using the CCK8 assay (Dojindo Molecular Technologies; Dojindo, Japan). Nonmalignant human breast (MCF10A) and vascular (HUVEC) cells were seeded onto 96-well plates at a seeding density of 25,000 cells/well. Imaging agent concentrations of 0.01, 0.05, and 0.10 mg/mL were incubated with the cells for 4, 24, and 48 h. Viability was measured relative to untreated cells. After incubation with nanoparticle-containing media, cells were incubated for 1-2 h with 10% CCK8 reagent in media according to the manufacturer's instructions. After incubation, the absorbance was measured at 450 nm using a Synergy HTX microplate reader (BioTek Instruments, Inc.; Winooski, VT) and relative viability was calculated.

### 2.8. Preparation of Tissue Phantoms with Cell-Based Tumor Inclusions

Tissue phantoms were prepared using previously published [5, 14, 24–26] and newly developed methods. Bovine liver and porcine muscle samples were obtained from a local grocery store and homogenized using a handheld homogenizing probe (VWR International; Radnor, PA). The sample volumes were recorded; then the samples were frozen and lyophilized for later use. Reconstituted phantoms were prepared by adding hemoglobin or tissue homogenate to gelatin and water. MDA-MB-231 cells were cultured and incubated with multimodal nanoparticle formulation for 12 h prior to imaging to simulate tumor uptake of SAMINs. Tumor-like inclusions were prepared by suspending the cells at 30 million cells/mL in a 5% alginate in PBS solution. Volumes of 5–50 *µ*L of cell suspension were pipetted into a solution of 100 mM CaCl_2_ and allowed to crosslink to form a spherical inclusion. Finally, the inclusions were placed in tissue phantom samples at different depths. Phantom samples were imaged with three imaging techniques relevant to integrating MR and fluorescence imaging. First, fluorescent images were obtained using a Pearl Trilogy Small Animal Imaging System (LI-COR; Lincoln, NE) utilizing the 800 nm excitation channel; then FIGS images were obtained using a 785 nm excitation source and integrated NIR and visible optical channels. Finally, the phantoms were imaged on a Bruker 7 T preclinical MRI scanner (*T*_1_-weighted MRI parameters for *T*_1_ mapping were: *T*_*R*_/*T*_*E*_, 800/6.38 ms; flip angle, 180°; field of view, 4 cm; slice thickness, 1.2 mm; matrix, 128 × 128).

### 2.9. Mouse Model of Breast Cancer

All animal studies were performed in accordance with a protocol approved by the UNMC Institutional Animal Care and Use Committee. Breast tumor xenografts were grown by injecting (how many) MDA-MB-231 tumor cells subcutaneously into 12-week-old female athymic nude mice (Jackson Laboratories; Bar Harbor, ME). When tumors reached 500–1000 mm^3^, mice were injected with contrast agent through intravenous infusion via a tail vein. Mice were dosed with SAMINs optimized to 0.005 mmol/kg Gd^3+^ and 10 nmol Cy7.5. For the control group, mice were dosed with 0.200 mmol/kg Magnevist. MRI scans of the mice were taken immediately prior to injection and then at 2, 4, and 24 h time points. *T*_1_-weighted MRI parameters for *T*_1_ mapping were *T*_*R*_/*T*_*E*_, 800/6.38 ms; flip angle, 180°; field of view, 4 cm; slice thickness, 1.2 mm; matrix, 128 × 128. After the final MR image acquisition, the mice were euthanized and imaged using FIGSS to simulate image-guided surgical removal of the malignancy. The mice were then dissected and organs removed to determine relative biodistribution studies as described below.

### 2.10. Relative Biodistribution of SAMINs

The relative biodistribution was characterized through fluorescence imaging and ICP-MS analysis. After imaging, mice were necropsied to image vital organs and tumors with a LI-COR Pearl Trilogy Small Animal NIR Imaging System. Images were processed in Image Studio software (LI-COR; Lincoln, NE). Organ samples were taken for each mouse and weighed, homogenized, and then prepared for ICP-MS analysis by digesting the samples with 1 : 3 nitric acid in hydrochloric acid solution. The ICP-MS biodistribution samples were then given to the UNMC nanomaterials core facility for instrumental analysis. Biodistribution was calculated from gadolinium content in the organ samples, and average organ weights for bone and muscle to calculate percent injected dose accumulation were obtained from the Jackson Laboratories online Mouse Phenome Database.

## 3. Results

### 3.1. Synthesis of Self-Assembled Multimodal Imaging Nanoparticles (SAMINs)

**Figure 1:**
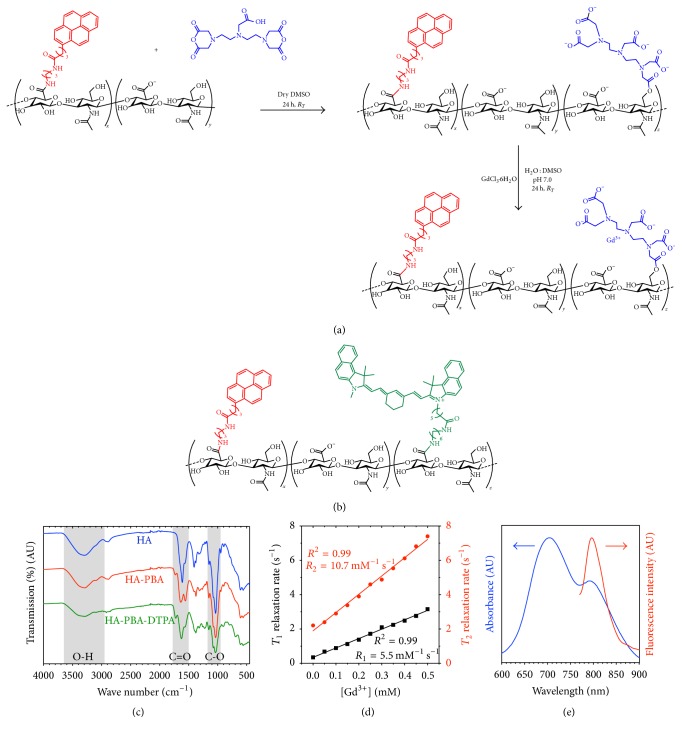
Synthesis of self-assembled multimodal imaging nanoparticles (SAMINs). (a) DTPA dianhydride is reacted with hydrophobically modified HA to produce HA-PBA-DPTA followed by Gd^3+^ complexation to yield paramagnetic HA-PBA-GdDTPA. (b) HA-PBA-Cy7.5 was synthesized as described in the corresponding text. (c) HA-PBA and HA-PBA-DTPA were confirmed with IR spectroscopy by an increase in C=O and O-H stretch peak intensities. (d) SAMIN relaxivity and (e) fluorescence were characterized and indicated the potential for magnetic resonant and optical imaging.

Self-assembled multimodal imaging nanoparticles (SAMINs) are comprised of two different types of modified HA: fluorescent HA and paramagnetic HA. Each conjugate is synthesized separately; then the ratio of fluorescent HA to paramagnetic HA is adjusted to reach optimal fluorescence and MR signal intensity as a contrast agent for integrated imaging. Synthesis and characterization of the Cy7.5-modified amphiphilic HA were performed as previously described; the Cy7.5 content was found to be 0.137 *µ*mol Cy7.5 per milligram of conjugate [5, 6]. Amphiphilic paramagnetic HA was synthesized as shown in Figure 1(a).  Figure 1(b) shows the structure of the fluorescent HA conjugate, HA-PBA-Cy7.5. Synthesis of HA-PBA-DTPA was confirmed by the presence of additional O-H stretch peak intensity and changes in the carbonyl peak, seen at 1500–1750 cm^−1^ in the infrared absorption spectrum shown in Figure 1(c). Synthesis of paramagnetic HA was confirmed by ICP-MS, and the gadolinium content was determined to be 0.215 *µ*mol per milligram conjugate. Relaxivity data of the SAMINs, shown in Figure 1(d), demonstrated *T*_1_ relaxivity of 5.5 mM^−1^s^−1^, which is comparable to that of Magnevist [23], a routinely used clinical MRI contrast agent. Fluorescent HA retains bright NIR fluorescence in the multimodal formulation (Figure 1(e)).

**Figure 2:**
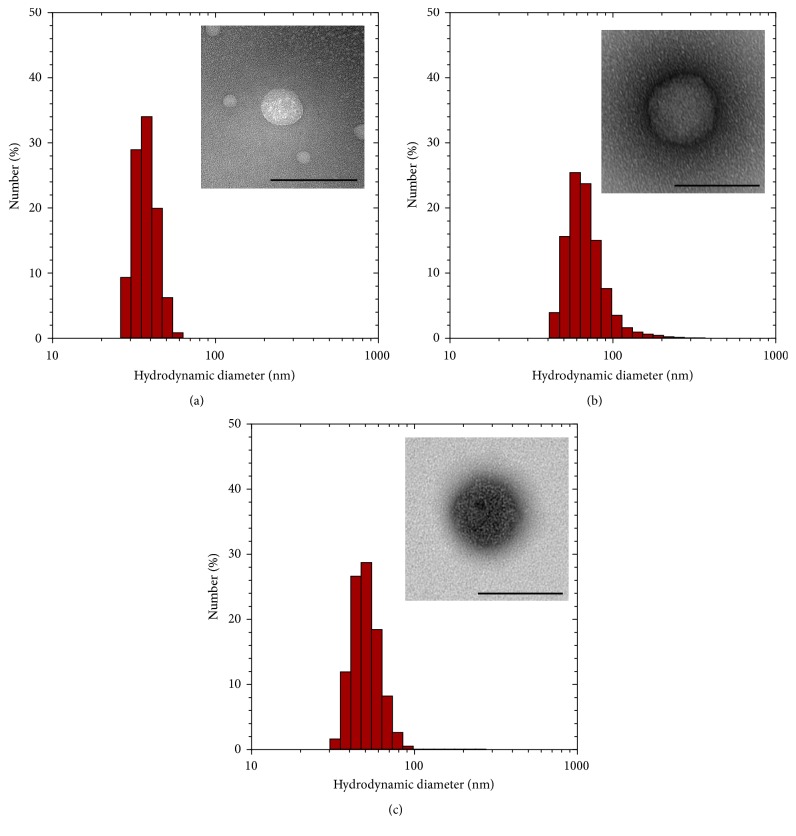
Physical characterization of nanoparticles from amphiphilic HA, including (a) HA-PBA-GdDTPA, (b) HA-PBA-Cy7.5, and (c) SAMINs. Histograms are dynamic light scattering data and inset images are TEM images of the same nanoparticles. The scale bar represents 100 nm.

Cy7.5-modified and gadolinium-modified amphiphilic HA conjugates and SAMINs result in nanoparticles as indicated in Figure 2. The nanoparticles comprised of paramagnetic HA have a smaller number average hydrodynamic, 69.57 nm with a PDI of 0.122, compared to HA-PBA-Cy7.5 or SAMINs. The HA-PBA-Cy7.5 nanoparticles had relatively higher number average hydrodynamic diameter of 92.82 nm with a PDI of 0.285. The SAMINs were measured to have a number average hydrodynamic diameter of 97.81 nm, with a PDI of 0.142. We expect that, due to the dynamic nature of polymeric aggregate nanoparticles, the different HA conjugates exchange between nanoparticles to form a homogeneous distribution of nanoparticles containing both species of HA conjugates. The zeta potential also differed between HA-PBA-Cy7.5 nanoparticles which had the lowest zeta potential (−12.3 mV), whereas paramagnetic HA nanoparticles showed a zeta potential of −7.81 mV and SAMINs demonstrated a −8.37 mV.

### 3.2. Formulation Optimization

**Figure 3:**
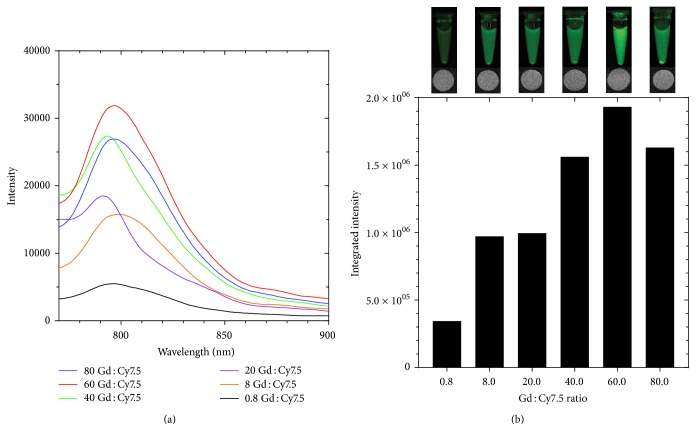
Ratio optimization of SAMINs. To achieve optimal contrast in both MR and fluorescent imaging modalities, the concentration of Cy7.5 must be calibrated to the ideal concentration of gadolinium for MRI contrast. (a) Fluorescence emission spectra of SAMINs with varying ratios of Gd : Cy7.5. The maximum fluorescence intensity as a function of concentration is shown in (b), from which the optimal ratio is derived. MR and NIR fluorescence images of the formulations are presented to illustrate the process of optimizing the ratio of gadolinium to Cy7.5.

The sensitivity of MRI using gadolinium as a contrast agent is lower than the sensitivity of fluorescence by several orders of magnitude [12]; therefore the ratio of gadolinium to Cy7.5 in SAMINs must be precisely tuned.  Figure 3 reports the results of imaging agent Gd : Cy7.5 ratio calibration in order to determine the optimal ratio for formulation for in vitro and in vivo studies. Upon higher Cy7.5 content, self-quenching is observed. The optimal Gd(III) : Cy7.5 ratio was found to be 60 Gd(III) : Cy7.5 molecules, as shown in Figures 3(a) and 3(b). Examining the integrated fluorescence intensities Figure 3(b), the 60 Gd(III) : Cy7.5 ratio was 20% brighter than the next closest ratio (80 Gd(III) : Cy7.5) and 460% brighter than the ratio at approximately 1 : 1 Gd(III) : Cy7.5. These results are further demonstrated visually through images of the vials containing the different formulations, and the 60 Gd : Cy7.5 sample shows the brightest fluorescence as seen in Figure 3(b), still maintaining consistent MR signal.

### 3.3. Cytotoxicity of SAMINs and Components

**Figure 4:**
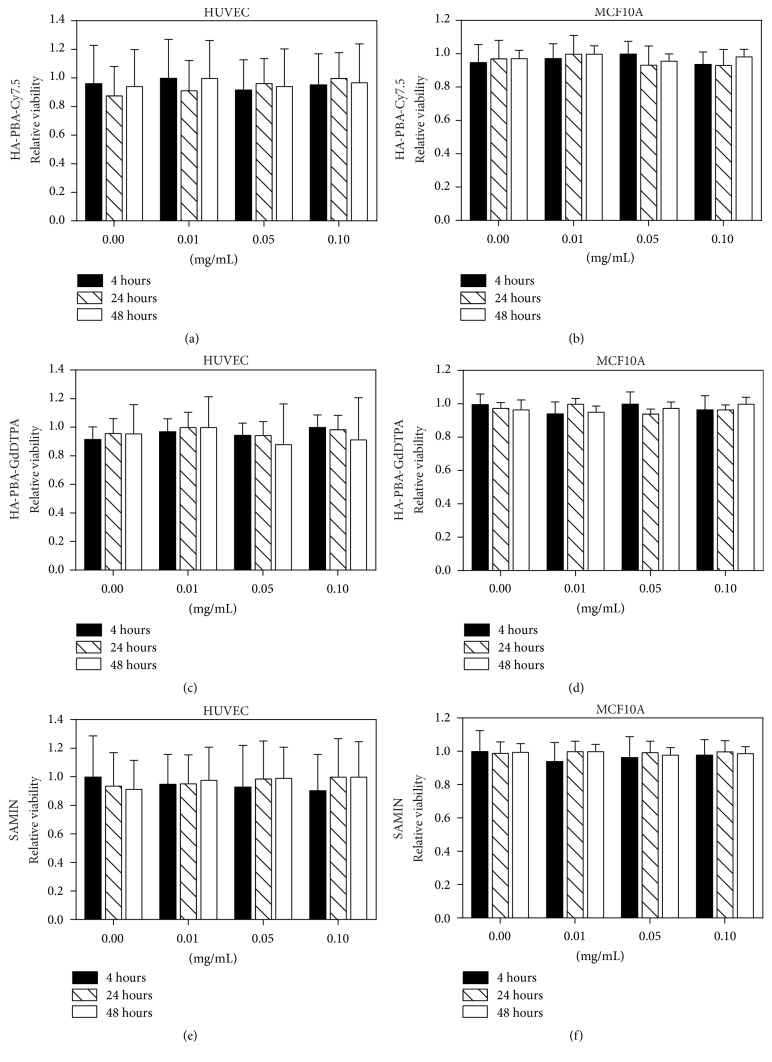
Cytotoxicity of SAMINs and individual components in two nonmalignant cell lines. The relative viability of each component formulation: ((a), (b)) HA-PBA-GdDTPA, ((c), (d)) HA-PBA- Cy7.5, and ((e), (f)) SAMINs were evaluated using a CCK8 cytotoxicity assay. (a), (c), (e) are data for HUVEC and (b), (d), (f) are for MCF10A cells.

Figure 4 shows the results of cell viability assays with two nonmalignant cell lines at three time points, demonstrating that SAMINs and both component HA derivatives are nontoxic at concentrations of 0.01, 0.05, and 0.10 mg/mL of contrast agent. MCF10A and HUVEC cells were chosen as cell lines to investigate if exposure to SAMINs resulted in any overall toxicity to representative, nonmalignant endothelial and epithelial cells, respectively. At a concentration of 0.10 mg/mL of contrast agent, HUVEC cells demonstrated 99.2% ± 21.4% viability with SAMINs, 97.0% ± 26.8% viability with HA-Cy7.5, and 91.4% ± 29.1% viability with HA-Gd. MCF10A cells exhibited a lower standard deviation than the HUVEC cells, with 98.8% ± 4.0% viability with SAMINs, 98.4% ± 4.2% viability with HA-Cy7.5, and 100.0% ± 3.9% viability with HA-Gd when treated with 0.10 mg/mL contrast agent.

### 3.4. Phantom Imaging Models

**Figure 5:**
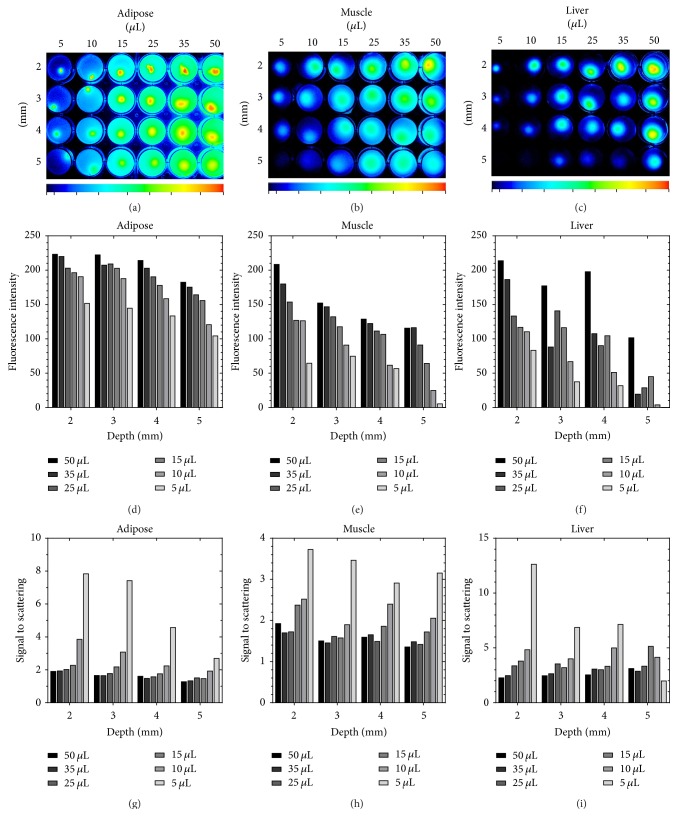
In vitro studies of depth detection and cellular uptake of SAMINs. Fluorescence contrast in three different simulated tissue phantom models demonstrates viability as a contrast agent in a variety of tissue types. (a) shows the contrast fluorescence image of tumor-like inclusions embedded into adipose tissue phantoms, which allows the depth-dependent signal (d) and scattering (g) profiles to be characterized. (b) shows the same data for simulated muscle tissue phantoms, which demonstrate different depth-dependent signal (e) and scattering (h) profiles. The fluorescence imaging data for liver tissue phantoms, for which depth-dependent signal (f) and scattering (i) profiles differ remarkably from both adipose and muscle tissue phantoms.

Figure 5 shows the results of fluorescence imaging using adipose, muscle, and liver tissue phantoms. The images of the different samples were analyzed to evaluate the difference in scattering and depth of detection of relevant tissue types with normalized signal intensities. Figures 5(d)–5(f) show the change in overall signal intensity over the region of interest for each depth and tissue type. The average fluorescence intensity per pixel was calculated to show differences in intensity by tissue type. Adipose tissue phantoms demonstrated the highest fluorescence intensity, due to less dense optical absorption. For example, 50 *µ*L inclusions at 5 mm deep in adipose were 60% brighter than muscle and 80% brighter than in the liver at the same depth. The same pattern of signal intensities is uniformly observed for each depth for adipose, muscle, and liver phantoms and is most apparent for the samples with 5 *µ*L inclusions. Adipose tissue phantoms demonstrated an average fluorescence intensity per pixel 18.5-fold greater for 5 *µ*L inclusion sample at 5 mm of deep in adipose compared to muscle; this size occlusion was only detectable to 4 mm in the liver phantom. Figures 5(g)–5(i) show the results of calculating the scattering versus signal intensity at different depths and sized for the tissue phantoms, which follow a trend inverse to that observed for mean fluorescence intensity per pixel. While the overall signal obtained from samples with smaller inclusion volume is lower, the signal to scattering ratio is higher. Furthermore, in samples with high optical density, a higher signal to scattering ratio was observed. Liver tissue phantom samples showed the highest signal to scattering ratios, with the 5 *µ*L inclusion at 2 mm showing a signal to scattering ratio of 12.64. Comparatively, the corresponding muscle tissue phantom (SSR = 3.73) and the corresponding adipose tissue phantom (SSR = 1.91) showed much lower SSR ratios.

### 3.5. MRI-Guided FIGS

**Figure 6:**
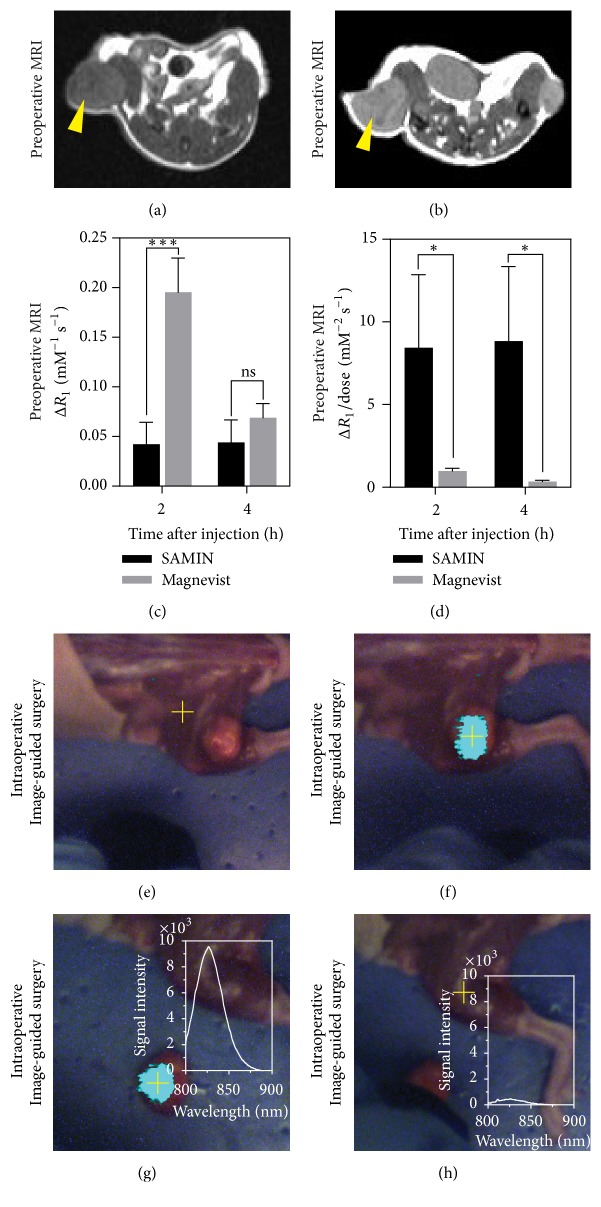
Integrated preoperative magnetic resonance imaging with intraoperative fluorescence image-guided surgery using a breast cancer xenograft model. (a) Preinjection image of mouse bearing breast cancer xenograft tumor denoted by yellow arrow. (b) Region of interest (tumor, yellow arrow) 24 h after IV injection of SAMINs (0.005 mmol/kg Gd^3+^; 0.5 *µ*mol/kg Cy7.5). (c) Change in relaxivity (*R*_1_) and change in *R*_1_ normalized to injected dose (d) after injection with Magnevist or SAMINS (for SAMINS, *N* = 6; for Magnevist, *N* = 3; *∗∗∗* denotes *p* < 0.001; *∗* denotes *p* < 0.05; ns denotes nonsignificant difference). ((e)–(h)) Fluorescence-guided surgery using SAMINs (laser excitation point denoted by yellow cross). (e) Excitation of tissue away from tumor indicates minimal signal, whereas (f) excitation of tumor shows strong NIR fluorescence signal due to SAMIN deposition in tumor. (g) Removed tumor was confirmed as well as the source of contrast enhancement, while (h) shows excitation of area from which the tumor removed is no longer fluorescent. The insets in (g), (h) show the NIR spectral response when the laser is direct on and off the contrast-enhanced areas, respectively.

Figure 6 shows the results of a proof-of-concept in vivo experiment using SAMINs to integrate preoperative MR imaging with fluorescence IGS. A representative contrast-enhanced MR image of a mouse bearing breast cancer xenografts is shown in Figures 6(a) and 6(b). The signal and contrast (relative to adjacent muscle) both increase due to the multimodal nanoparticles (Figures 6(c) and 6(d)), where the *R*_1_ enhancement when normalized for dosage is found to be significantly higher for the SAMINs over Magnevist. While Magnevist may show higher change in *R*_1_, the SAMIN formulation results in a better signal increase with a 40x lower dosage of gadolinium. After MR imaging, the mouse was euthanized and underwent a mock surgical resection of the MR contrast-enhanced tumor using fluorescence contrast-enhanced IGS. Representative fluorescence images are shown in Figures 6(e)–6(h). The IGS system utilizes a handheld spectroscopic “pen” that uses a laser for excitation. When the pen excites tissue just off the tumor (Figure 6(e)), no contrast enhancement is observed in the wide-field surgical imaging display. When the pen is moved onto the tumor, strong NIR signal due to Cy7.5 is observed in the NIR channel of the IGS system, pseudocolored cyan, and overlaid onto the visible light channel of the system (Figure 6(f)). The tumor was then resected using fluorescence image-guidance. Figure 6(f) shows that the tumor was causing the fluorescence emission, while the connecting muscle adjacent to the xenograft resulted in background levels of fluorescence (Figure 6(h)). Inset images in Figures 6(g) and 6(h) report the NIR spectroscopic signal from Cy7.5 contained in the SAMINs and correspond to the MR signal and fluorescence IGS.

### 3.6. Biodistribution of SAMINs

**Figure 7:**
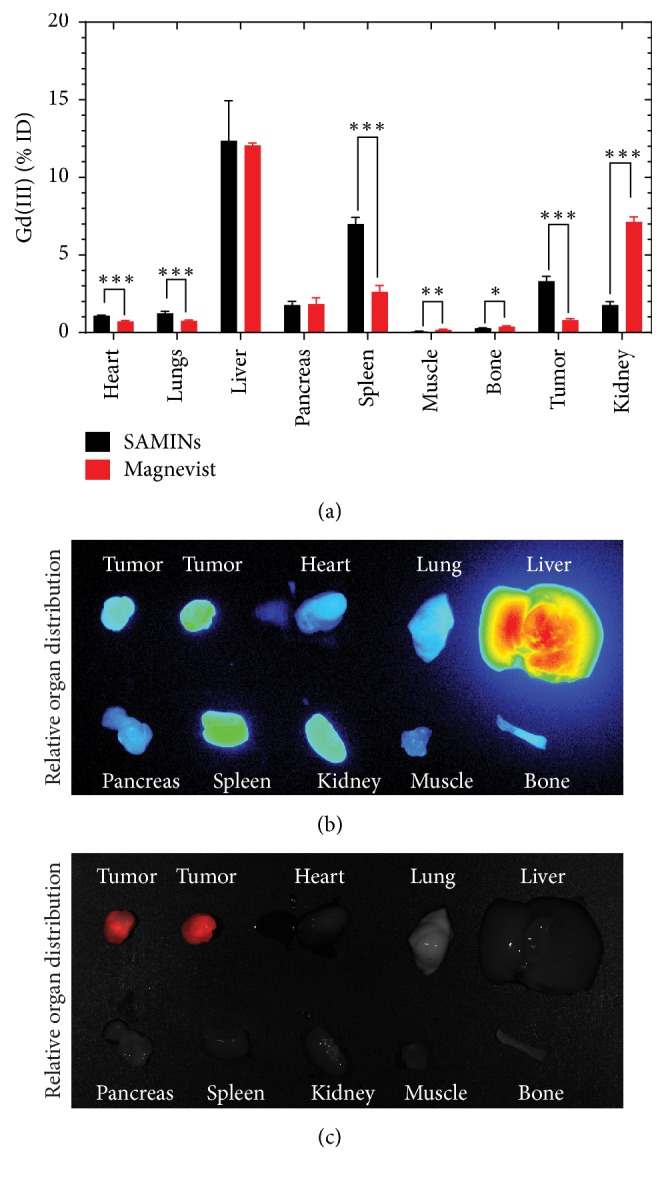
Biodistribution of multimodal imaging agents. (a) Comparative biodistribution of gadolinium when dosed as SAMINs versus Magnevist (for *∗∗∗*, *p* < 0.001; *∗∗*, *p* < 0.01; *∗*, *p* < 0.05). (b) Relative distribution of SAMINs as NIR fluorescence signal indicates increase in signal intensity in tumor (confirmed by iRFP expression from the MDA-MB-231 cells in (c)) and clearance organs.

Figure 7 reports the biodistribution of the SAMINs with Magnevist as a control. Percent injected dose of gadolinium was obtained via ICP-MS analysis of organ samples as shown in Figure 7(a). Only the liver and pancreas show insignificant differences between the SAMINs and Magnevist, while significant difference is observed in all other organs of interest. Where SAMINs yielded a 3.30% injected dose of gadolinium accumulation in the tumor, the Magnevist showed only 0.80% accumulation in the tumor. Furthermore, the SAMIN formulation leads to lower accumulation of gadolinium in the muscle tissue than Magnevist, resulting in higher tumor-muscle contrast as also demonstrated in Figure 6(a). Figures 7(b) and 7(c) confirm that NIR signal is consistent with the presence of tumor (iRFP fluorescence in Figure 7(c)) and clearance organs.

## 4. Discussion

We demonstrate the use of self-assembled multimodal imaging nanoparticles (SAMINs) to integrate preoperative MR imaging with fluorescence image-guided surgery. We successfully synthesized new contrast agents, which provide both fluorescence and MR signal in vivo and in vitro. Using a mixed micelle formulation provides a way to convenient and reproducible method to optimize the ratio of MR to fluorescence component in a nanoparticle formulation.

The synthesis of paramagnetic HA introduced new considerations in formulation. Prior work with gadolinium-modified HA has only used HA as a macromolecular scaffold rather than an amphiphile [19, 27], where the polymers do not self-assemble into nanoparticles. In this work, as well as our prior work with HA-based imaging [5, 6, 14] and drug delivery agents [15], we modify HA with hydrophobic moieties (specifically PBA) to drive self-assembly into nanoparticles. However, the addition of gadolinium was found to increase hydrophobicity, requiring the percent weight composition of hydrophobic PBA to be lower on paramagnetic HA than on fluorescent HA. One of the distinct advantages of using a mixed micelle formulation, as opposed to adding both gadolinium and Cy7.5 to the same strand, was the ability to calibrate the hydrophobicity of the HA derivatives. Previous work by others [28, 29] has demonstrated the use of dual-functionalized polymeric contrast agents, but in such formulations the ability to calibrate contrast agent ratio or hydrophobicity is much less apparent.

Prior to moving into more in-depth studies, the cytotoxicity of the SAMINs needed to be assessed. In our studies, each component was tested individually to ensure that the HA-PBA-Cy7.5 and HA-PBA-GdDTPA mixed micelle components were cytotoxic, and then the multimodal formulation was tested as well. The concentrations of 0.01 mg/mL, 0.05 mg/mL, and 0.10 mg/mL were chosen to be physiologically relevant from our biodistribution results and are consistent with our previous work [6]. Gadolinium ions, when not bound to a chelating ligand such as DTPA, are known to be highly toxic and therefore any agent bearing gadolinium must be shown to be nontoxic at therapeutic dosage [12, 23]. Many other formulations based on inorganic nanoparticles suffer from toxicity concerns [12], and while iron oxide nanoparticles have recently emerged as a strategy for MR contrast and are less toxic, iron oxide-based agents are primarily used for *T*_2_ contrast [30]. In our experiments, the gadolinium-bearing HA conjugates are shown to have low cytotoxicity while maintaining high *T*_1_ contrast, an advantage over other inorganic MR contrast agents.

When evaluating the efficacy of experimental contrast agents, sample to sample variation can be difficult to minimize [31]. Cell assays help to characterize uptake in a more controlled manner but are limited in scope. As shown by our lab [14] and others [25, 26, 31–33], the use of tissue-mimicking phantoms can help to characterize optical properties and potential for contrast in a highly reproducible manner. Developing these models for different tissues aids in simulating the biological conditions observed in vivo; in this work, we expand our use of simulated tissue phantoms to model liver and muscle tissue and demonstrate the use of cell-based inclusions to model tumors. While the tumor microenvironment is difficult to model, the use of cell-based inclusions demonstrates the possible outcome of imaging in vivo using the same cancer cell lines and provides an insightful step of imaging agent evaluation prior to further in vivo studies.

In addition to characterizing uptake and overall signal, the use of tissue-mimicking phantoms allows investigation into the ability to detect tumors that may be small or located deeper in tissue. Furthermore, many cancers metastasize to different organs and tissues throughout the body, which have inherently different optical profiles. Fluorescence imaging has limitations due to depth and difference in scattering profiles based on the organ, and the use of tissue phantoms allows characterization of these effects in a controlled manner. Higher scattering is likely a function of the increased content of contrast agent, resulting in higher signal in all directions and therefor scattering through the phantom media. The insights gained from these experiments help to predict the signal intensity that will be obtained from in vivo samples, ultimately guiding the design of new contrast agents.

After validation in tissue phantom models, we performed a study to analyze the efficacy of our contrast agents in a mouse model of breast cancer. However, the use of polymeric agents for gadolinium delivery remains difficult due to the concentration of gadolinium needed to deliver high contrast. A limiting factor in the dosage of macromolecular gadolinium-bearing contrast agents is the overall mass of sample required to achieve adequate gadolinium concentration [34–36]. Since the mass percent of gadolinium in these contrast agents is relatively low, dosing is a unique challenge as opposed to other contrast agents such as inorganic nanoparticles or small molecule contrast agents. However, our results concur with previously published data [19, 27, 37–39] for the use of macromolecular contrast agents, in which MR signal is shown to increase and provide adequate contrast in vivo. The contrast obtained in MR imaging is shown to increase over a 24 h period, which is also consistent with our previously published data [5] on the biodistribution and optimal imaging time for fluorescent HA in IGS.

After preoperative MRI, the fluorescence imaging performed with IGS shows high contrast in the tumors. When compared to the muscle tissue, the tumor demonstrates much higher signal than the muscle. While inorganic multimodal nanoparticle contrast agents have been shown to be effective for both fluorescence and MR imaging [40–44], the reduced cytotoxicity of our formulation and the easily calibrated contrast agent ratio provide key advantages over the existing technology. Other groups have used polymers such as poly(ethylene glycol) [39, 45, 46]; however the use of naturally occurring biopolymers such as hyaluronic acid is gaining prevalence [47–49] of use in nanomedicine for better biocompatibility [50] and targeting [47]. The enhanced targeting for certain tumors achieved using hyaluronic acid as the backbone for our contrast agents ensures specific uptake, resulting in higher contrast, as demonstrated in the ex vivo fluorescence imaging of the organs.

The in vivo contrast enhancement using the SAMINs is confirmed through biodistribution studies with ICP-MS. We have previously reported on the biodistribution of Cy7.5-labeled HA versus free Cy7.5 [5, 6], and the results obtained from gadolinium biodistribution concur with the previous observations of increased tumor accumulation and higher tumor-muscle contrast. Although the dosage of gadolinium was 40 times less (0.005 mmol/kg) than the dosage of gadolinium with Magnevist (0.200 mmol/kg), the tumor-muscle contrast was obtained using the SAMIN formulation and could result in improved imaging capabilities. The SAMIN formulation also resulted in higher uptake in the spleen, while Magnevist showed higher accumulation in the kidney, exhibiting renal clearance.

## 5. Conclusions

Improving the contrast between healthy, noncancerous tissue, and malignant tissue remains the top priority for research in image-guided surgery, including when evaluating preoperative procedures. In this work, we have developed a nanoparticle formulation capable of providing contrast for both MRI and FIGS, aimed at improving surgical guidance. Further work will aim at increasing the MR signal obtained from the nanoparticles, as the current mass of paramagnetic polymeric conjugate required to achieve sufficient contrast is a limitation to increasing the dosage. In conclusion, this work is a starting point for the development of improved contrast agents to leverage the targetability, improved biodistribution, and biocompatibility of polymeric nanomedicine with the versatility of MRI and the sensitivity of FIGS.
